# *Pterygodermatites* (*Paucipectines*) *baiomydis* n. sp. (Nematoda: Rictulariidae), a parasite of *Baiomys taylori* (Cricetidae)

**DOI:** 10.1051/parasite/2014057

**Published:** 2014-11-07

**Authors:** Christina Lynggaard, Luis García-Prieto, Carmen Guzmán-Cornejo, David Osorio-Sarabia

**Affiliations:** 1 Laboratorio de Helmintología, Instituto de Biología, Universidad Nacional Autónoma de México, Apartado Postal 70–153 CP 04510 México, D.F. Mexico; 2 Laboratorio de Acarología, Facultad de Ciencias, Universidad Nacional Autónoma de México CP 04510 México, D.F. Mexico

**Keywords:** Nematoda, *Pterygodermatites*, *Baiomys taylori*, Rodentia, Cricetidae, Mexico

## Abstract

*Pterygodermatites* (*Paucipectines*) *baiomydis* n. sp., an intestinal parasite of the northern pygmy mouse, *Baiomys taylori* (Cricetidae), collected in La Yerbabuena, Colima, Mexico, is described herein. Specimens were studied using light and scanning electronic microscopy. This is the 19th species of the subgenus *Paucipectines* described worldwide and the fourth collected in Mexico. It is differentiated from the remaining species in the subgenus by having 25 perioral denticles, arranged in a triangle (seven on each lateroventral margin, and eleven on the dorsal margin), and 10 pairs of caudal papillae.

## Abstract (fr)

*Pterygodermatites* (*Paucipectines*) *baiomydis* n. sp., parasite intestinal du rongeur *Baiomys taylori* (Cricetidae), collecté dans La Yerbabuena, Colima, Mexique, est décrit. Les échantillons ont été étudiés en utilisant microscopie photonique et microscopie électronique à balayage. Ceci est la dix-neuvième espèce du sous-genre *Paucipectines* décrite dans le monde et la quatrième au Mexique. Elle se distingue des autres espèces dans le sous-genre par la possession de 25 denticules périoraux disposés en triangle (sept sur chaque marge latéroventrale et onze sur la marge dorsale) et dix paires de papilles caudales.

## Introduction

Cricetid rodents are one of the most speciose groups of mammals of the New World with approximately 600 species [25]. From the 141 species known to occur in Mexico [25], only 25 had been examined for helminths prior to this study. These surveys resulted in the inventory of 45 species of helminths [5].

As part of an ongoing project to describe the metazoan fauna associated with rodents from Mexico, we analyzed 27 cricetid taxa in seven localities from the Mexican states of Colima, Guerrero, Jalisco, Michoacán, and Oaxaca. The main goal of this paper is to describe a new species of the nematode subgenus *Pterygodermatites* (*Paucipectines*) Quentin, 1969, as a parasite of the northern pygmy mouse *Baiomys taylori* (Thomas, 1887) in Colima, Mexico.

## Materials and methods

In December, 2011, two specimens of *B. taylori* were collected in La Yerbabuena (19°28′39′′ N, 103°40′46′′ W) in Comala, Colima, Mexico. Hosts were collected with permission (FAUT-0170), issued by Secretaría del Medio Ambiente y Recursos Naturales, Mexico. Rodents were anesthetized by isoflurane inhalation, euthanized by cervical dislocation, and examined for helminth parasites. Helminths were removed from the intestine and placed in 0.85% saline solution, fixed in hot 4% formaldehyde, and stored in 80% ethanol. Nematodes were cleared with Amman’s lactophenol and temporarily mounted for morphological study. Population parameters follow Bush et al. [1]. Measurements, expressed in micrometres unless otherwise stated, are given as the range, followed by mean, standard deviation, and sample size in parentheses. Figures were drawn with the aid of a drawing tube. Specimens for scanning electron microscopy were dehydrated in a graded ethanol series, critical-point dried with CO_2_, and then coated with a gold-palladium mixture. Specimens were examined with a Hitachi SU1510 electron microscope. Type specimens were deposited at the Colección Nacional de Helmintos (CNHE), Instituto de Biología, Universidad Nacional Autónoma de México, Mexico City, Mexico.

## *Pterygodermatites* (*Paucipectines*) *baiomydis* n. sp.

urn:lsid:zoobank.org:act:6B93D4D1-44B0-44C4-80CC-0F8018B9E950

(Figs. 1A–1K)

**Figure 1.**
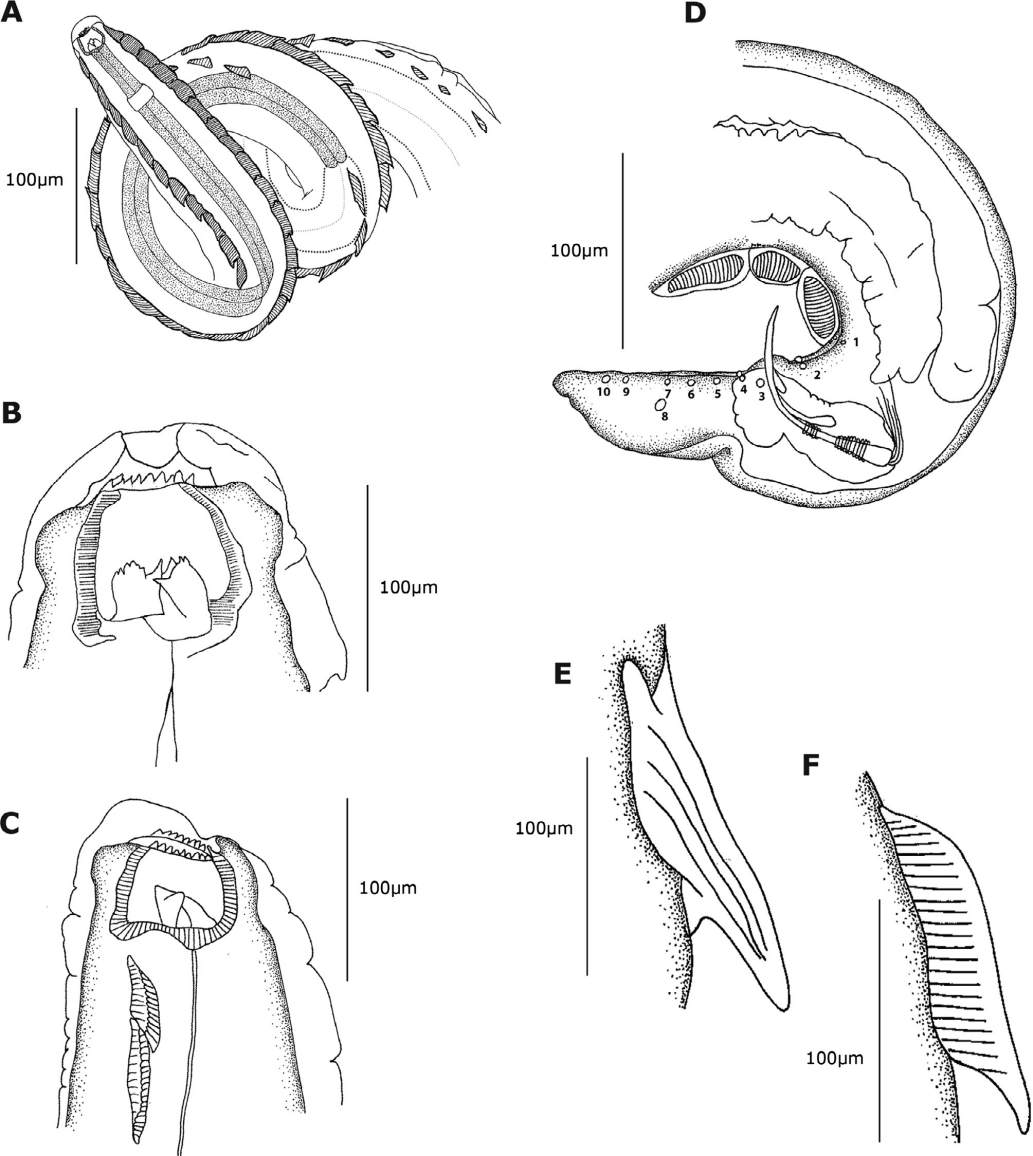
(A–F) *Pterygodermatites* (*Paucipectines*) *baiomydis* n. sp., a parasite of *Baiomys taylori* from Colima, Mexico. **A.** Female, anterior region. **B.** Female, cephalic end, dorsal view. **C.** Cephalic end, lateral view. **D.** Male, caudal region, lateral view. Caudal papillae numbered. **E.** Spine, lateral view. **F.** Comb, lateral view.

**Figure 2.**
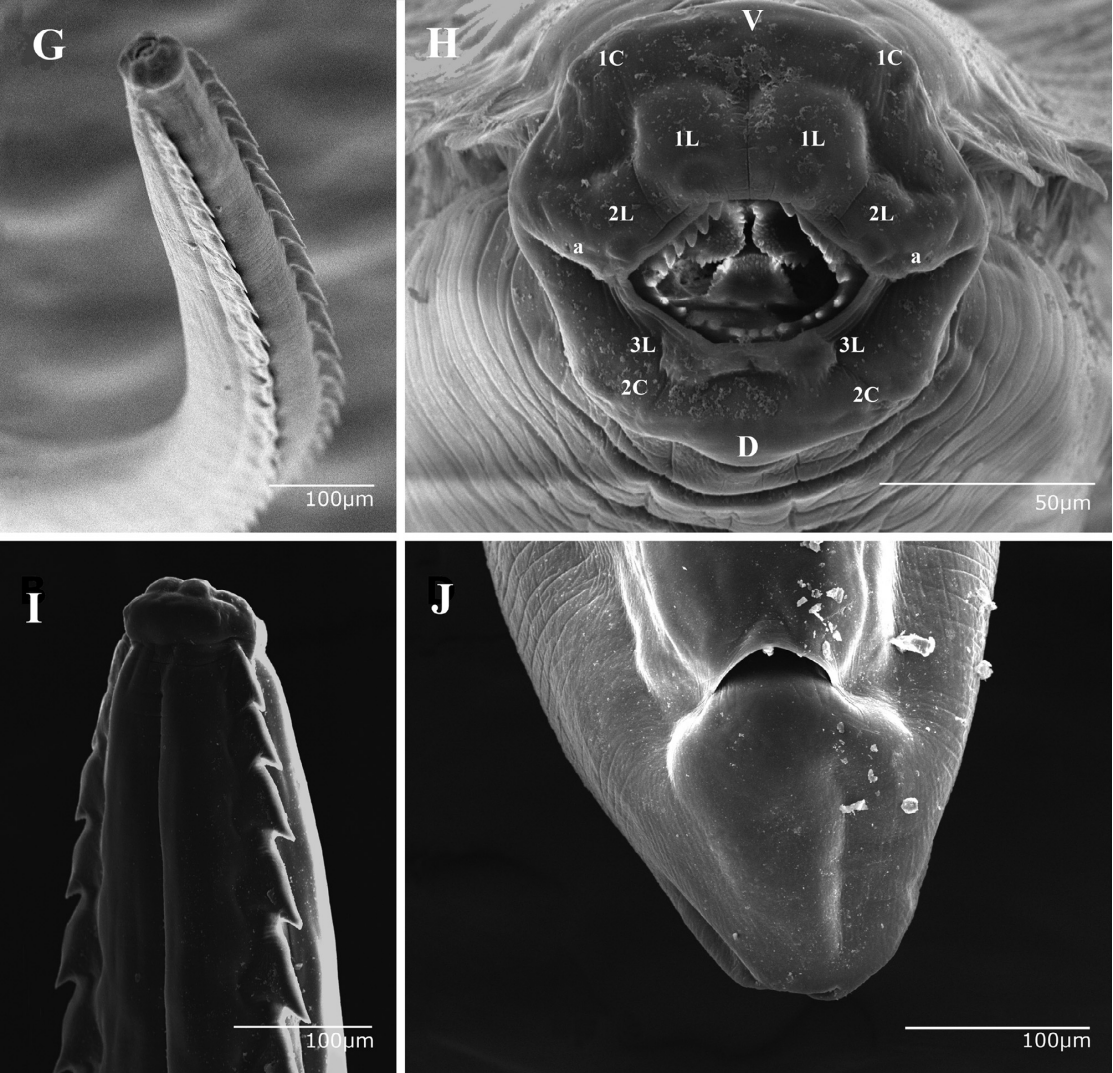
(G–J) *Pterygodermatites* (*Paucipectines*) *baiomydis* n. sp., a parasite of *Baiomys taylori* from Colima, Mexico. Scanning electron micrographs: **G.** Female, anterior end, subventral combs. **H.** Female, anterior end, apical view [Cephalic papillae: **1C**. Pair 1. **2C**. Pair 2. Labial papillae: **1Iv**. Internal pair 1 (ventral). **2Ilv**. Internal pair 2 (lateroventral). **3Ild**. Internal pair 3 (dorsal). **a**. Amphid. **V.** Ventral region. **D.** Dorsal region]. **I.** Female, anterior end, ventral view. **J.** Female, posterior end, ventral view.

Type host: *Baiomys taylori* (Thomas, 1887) (Rodentia: Cricetidae). Symbiotype deposited at Museo de Zoología Alfonso L. Herrera, Facultad de Ciencias (MZFC-M) de la Universidad Nacional Autónoma de México, Mexico City, Mexico (MZFC-M 11988, 12294). Date of collection: December, 2011.

Type locality: La Yerbabuena (19°37′26.36′′ N, 103°32′41.20′′ W), Colima, Mexico.

Site of infection: Intestine.

Prevalence and intensity of infection: two of two hosts examined were infected; mean intensity 3.5, range 1–7.

Etymology: The species is named after the host’s generic name.

Specimens deposited: Holotype: CNHE 8662 (female); paratypes CNHE 8663 (1 male) and CNHE 8664 (5 females).

### Description

#### General

Medium-sized nematodes, body slightly widened at posterior end, with thick cuticle. Oral opening apical, surrounded by 5 pairs of papillae, 2 cephalic pairs, and 3 internal labial pairs (1 dorsal, 1 ventral and 1 lateroventral pair). Amphids lateral. Oral opening asymmetric, somewhat hexagonal, with thick margins. Seven denticles on lateroventral margins, and 11 denticles on dorsal margin; 3 internal pharyngeal teeth at bottom of buccal capsule, 1 dorsal and 2 lateroventral. Two subventral rows of cuticular projections along body, starting at end of buccal capsule and ending at anus in females and at level of ventral fans in males. A single type of cuticular projection present in males (combs), and 2 different types in females: simple spines and combs.

Esophagus divided into short muscular and long glandular portions.

#### Male (based on the paratype)

**Figure 3.**
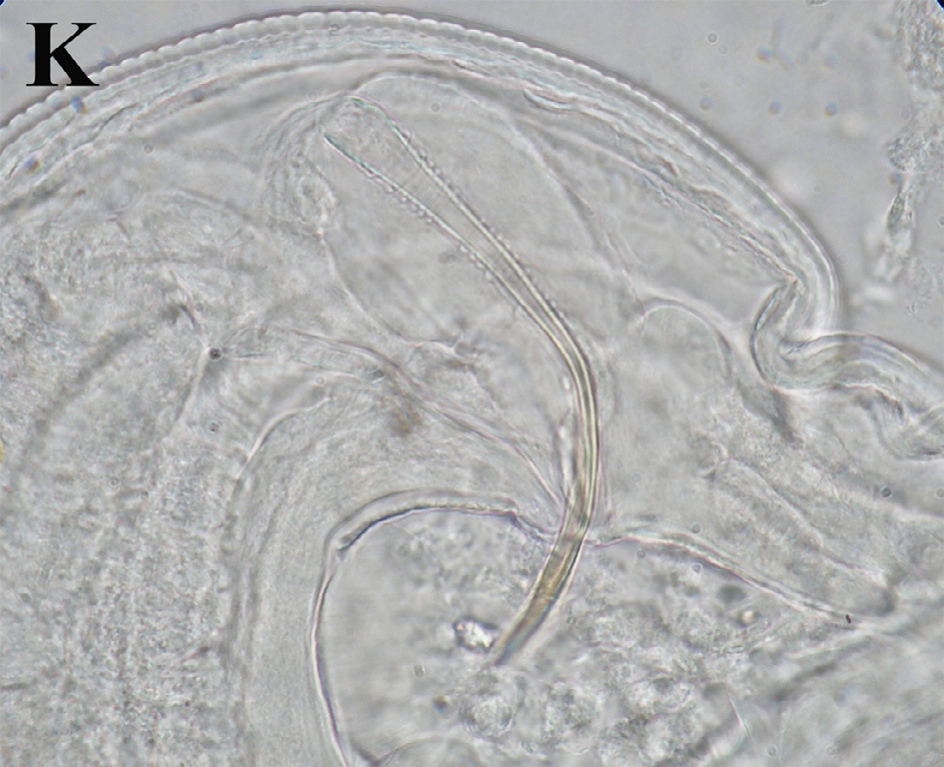
(K) *Pterygodermatites* (*Paucipectines*) *baiomydis* n. sp., a parasite of *Baiomys taylori* from Colima, Mexico. **K.** Left spicule surrounded by folded spicular sheath.

Total length 2.93 mm, width at base of buccal capsule 0.1. Buccal cavity 27 deep by 45 wide in lateral view. Number of perioral denticles undetermined. Three esophageal teeth, 10 long. Esophagus not measured. Nerve ring located 210 from anterior end. Deirids and excretory pore not observed. Forty-two pairs of subventral combs, 32–89 (69 ± 18, *n* = 10) long, and three ventral cuticular fans anterior to cloaca (*sensu* Lichtenfels [8]). Anterior fan 47 long, second and third fan, 30 long. Tail length 110. Spicules markedly unequal, curved ventrally, with blunt tips; left 110 long (Fig. 3K); right spicule simple, 47 long. Gubernaculum 10 long. Ten pairs of caudal papillae: 2 precloacal pairs, 1 at distal base of third fan and 1 between third fan and cloaca; 8 postcloacal pairs: 1 sublateral pair near cloaca, followed by 4 subventral pairs and 1 sublateral pair, the latter at same distance from cloaca as the last subventral pair; the last 2 pairs subventral and subterminal (Fig. 1D). Phasmids posterior to caudal papillae.

#### Female (based on the holotype and five paratypes)

Total length 18.35–21.41 (20.06 ± 1.4, *n* = 5) mm, prevulvar width 210–310 (280 ± 40, *n* = 5), postvulvar width 300–450 (340 ± 60, *n* = 5). Buccal cavity 90–120 (100 ± 23, *n* = 5) deep by 70–110 (100 ± 14, *n* = 5) wide in lateral view (Fig. 1C). Twenty-five perioral denticles 6.51–9.76 (7.80 ± 1, *n* = 25) long, arranged in triangle (7 on each lateroventral margin, and 11 on dorsal margin; Fig. 2H). Three esophageal teeth 20–40 (30 ± 5, *n* = 15) long (Fig. 1B). Total length of esophagus 2260–2650 (2470 ± 140, *n* = 5) (13.6% of body length), muscular portion 310–440 (380 ± 70, *n* = 3) long, 50–70 (60 ± 8, *n* = 3) wide at level of nerve ring; glandular portion 1630–1860 (1710 ± 130, *n* = 3) long, 80–110 (94 ± 10, *n* = 3) wide. Nerve ring and deirids located 210–350 (280 ± 70, *n* = 5) and 460–660 (560 ± 100, *n* = 4) from anterior end, respectively (Fig. 1A). Excretory pore not observed. Vulva 2220–3950 (3420 ± 780, *n* = 5) from anterior end and 770–1830 (1190 ± 410, *n* = 5) from esophago-intestinal junction, at level of the 42nd cuticular projection. Two subventral rows with 64–75 (71 ± 3.54, *n* = 5) cuticular projections (Figs. 2G and 2H); 38–44 (42 ± 2.53, *n* = 5) prevulvar combs (Fig. 1F) and 25–31 (29 ± 2.22, *n* = 5) postvulvar spines (Fig. 1E); –16 (12 ± 2.13, *n* = 5) combs between posterior end of esophagus and vulva. Maximum length of combs and spines 100–220 (160 ± 50, *n* = 5) and 130–240 (170 ± 40, *n* = 5), respectively. Last spine located 1020–2108 (1632 ± 420, *n* = 5) from posterior end. Tail 160–250 (210 ± 20, *n* = 5) long (Fig. 2J). Larvated eggs 30–40 (31 ± 4, *n* = 10) by 15–34 (25 ± 4, *n* = 10).

### Remarks

The morphology of the buccal capsule of the specimens described in this study, particularly the apical position of the oral opening, enables us to include them in the subgenus *Pterygodermatites* (*Paucipectines*) [3]. However, the number of prevulvar cuticular processes in *P.* (*Paucipectines*) *baiomydis* n. sp. (38–44) differs from the range described for the subgenus (29–39) by Quentin [19]. Because variations of this trait have been reported for other species included in the subgenus, for example *P.* (*P*.) *kozeki* (Chabaud and Bain, 1981), *P.* (*P*.) *jagerskioldi* (Lent and Freitas, 1935), and *P.* (*P*.) *dipodomis* (Tiner, 1948), all possessing more than 39 prevulvar cuticular processes, the diagnosis of this subgenus should be amended (Table 1).

**Table 1.** Selected features of the species included in the subgenus *Pterygodermatites* (*Paucipectines*) worldwide.

Distribution/species/(host family)	Shape of buccal capsule	DN male*	DN female*	No. of prevulvar combs/total	No. of combs in male	No. of fans	Spicule size left/right	CP: p/u†	Last spine-tail tip distance	Vulva-esophago-intestinal junction distance	Reference
Argentina											
*P. azarai* (Cricetidae)	Trapezoidal	17–19	17–19	30–31/67–71	39–43	3–4	80/60	10/1	–	1210	[22]
*P. chaetophracti* (Dasypodidae)	Trapezoidal	16–18	18	–/62–67	41	3	130/50	8/0	900–920	1215	[13, 15]
*P. massoiai* (Cricetidae)	–	–	Numerous	39/76	–	–	–	–	108	900	[21]
*P. spinicaudatis* (Microbiotheriidae)	Triangular	11–12	11–12	36/68–69	43	4	120/50	–	–	At junction or immediately posterior to it	[16]
Brazil											
*P. elegans* (Molossidae Didelphidae)	–	–	–	–	–	0	110/50	8/2	–	800	[24]
*P. jagerskioldi* (Didelphidae)	Trapezoidal	–	16	36–40/80	–	–	–	–	660–1070	Anterior to posterior end of esophagus	[10]
*P. zygodontomys* (Cricetidae)	Triangular	17	21	38/81	41	3	100/50	10/1	770	730	[18]
*P. hymanae* (Didelphidae)	Triangular	14	14	35/63	42	3	80–90/40–50	8/0	–	680	[7]
Colombia											
*P. kozeki* (Didelphidae)	Triangular	14–20	14–20	40/65–67	41	1	250	10/0	–	700–.900	[14]
Mexico											
*P. bayomydis* (Cricetidae)	Hexagonal	25	25	38–44/71	42	3	110/50	10/0	1020–2108	770–1830	Present study
Russia											
*P. baicalensis* (Muridae)	–	–	18 Bicuspid	31/62	42	3	260/130	9/0	–	1570	[20]
*P. sibiricensis* (Cricetidae)	Rectangular	19–24	19–24	33–34/61–67	44	3	110/53	7/1	–	Posterior to junction	[12]
USA											
*P. coloradensis* (Cricetidae, Sciuridae)	Oblong	–	17	32–34/65	42	0	240/200	10/1	–	At junction	[9]
*P. dipodomis* (Heteromyidae)	Rounded	–	18	40/71–74	38–40	3	100–110/50	9/0	2150	580	[17, 23]
*P. microti* (Cricetidae)	Rounded	26	24–25	32–33/64–66	45	0–1	Equal	10/0	460–680	850–1320	[11]
*P. ondatrae* (Cricetidae)	Rounded	–	18	32/73–75	52	1	110/100	4/1	2500–3000	500–1000	[2]
*P. onychomis* (Cricetidae)	Rounded	–	26	32/56–60	–	–	–	–	–	Anterior to posterior end of esophagus	[4]
*P. parkeri* (Cricetidae, Dipodidae, Sciuridae)	Oval	13	14–19	30–31/61–67	42	0	270/260	10/1	–	Posterior to end of esophagus	[9]
*P. peromysci* (Cricetidae, Sciuridae)	Oval	12	16–19	29/61–64	41	3	90–10/40–50	10/1	–	Anterior to posterior end of esophagus	[9]

* DN = Number of denticles.

† CP: p/u = Caudal papillae: paired/unpaired.

To date, 18 species of this subgenus have been described as parasites of rodents (12 species), armadillos (1 species), bats (1 species), and marsupials (4 species) (Table 1). *Pterygodermatites* (*P*.) *baiomydis* most closely resembles *P*. (*P*.) *dipodomis* and *P*. (*P*.) *zygodontomys* (Quentin, 1967). All three species share traits such as the number of prevulvar combs and fans, unequal spicules, and all three parasitize rodents (Table 1). However, the number of denticles is greater in *P*. (*P*.) *baiomydis* (25 vs. 18 and 17–21, respectively); the distance from the last spine to the tip of the tail in females of *P*. (*P*.) *zygodontomys* is smaller (770), and the distance from the vulva to the esophago-intestinal junction in *P.* (*P*.) *baiomydis* is greater (1190) than in the other two species (730 and 580, respectively). *Pterygodermatites* (*P.*) *zygodontomys* features an unpaired papilla, which makes it different from *P.* (*P*.) *baiomydis.* The new species differs from *P*. (*P*.) *dipodomis* by the rounded oral opening, and the smaller number of caudal papillae (9 vs. 10).

In addition, *P.* (*P*.) *baiomydis* can be differentiated from *P.* (*P*.) *elegans* (Travassos, 1928), *P.* (*P*.) *coloradensis* (Hall, 1916) [6], and *P.* (*P*.) *parkeri* Lichtenfels, 1970, because these three species lack fans and have unpaired caudal papillae, whereas the new species has three fans and all papillae are paired. From *P.* (*P*.) *hymanae* Jiménez and Patterson, 2012, *P.* (*P*.) *jagerskioldi*, and *P.* (*P*.) *spinicaudatis* Navone and Suriano, 1992, the new species is distinguished based on the number of perioral denticles (14, 16, and 11–12, respectively vs. 25 in *P.* (*P*.) *baiomydis*), as well as the host group (marsupials vs. rodents) and distribution (South America vs. North America). The number of fans and spicule size allow us to differentiate *P.* (*P*.) *kozeki*, *P.* (*P*.) *ondatrae* (Chandler, 1941) and *P.* (*P*.) *microti* (McPherson and Tiner, 1952) from the new species, since these three species have only one fan (instead of three fans as in the new species) and their spicules are almost equal in size, whereas in *P.* (*P*.) *baiomydis* spicules are unequal. Two other species, *P.* (*P*.) *peromysci* Lichtenfels, 1970 and *P.* (*P*.) *onychomis* (Cuckler, 1939), differ from the new species by the position of the vulva, which in those species is anterior to the esophago-intestinal junction, whereas in *P*. (*P*.) *baiomydis* it is situated 1190 posterior to the esophago-intestinal junction. Moreover, the oral opening in *P.* (*P*.) *peromysci* and *P.* (*P*.) *onychomis* is a rounded oval, but hexagonal in the new species. In having fewer denticles, arranged in a trapezoid, *P.* (*P*.) *chaetophracti* (Navone and Lombardero, 1980) and *P.* (*P*.) *azarai* (Sutton, 1984) can be distinguished from the Mexican species (16–18 and 17–19, respectively vs. 25 disposed in a hexagon).

For most of the species of the subgenus, males are unknown or insufficiently described; for that reason, female morphological traits are commonly used in species differentiation, partially solving this problem. However, in species such as *P.* (*P*.) *massoiai* (Sutton, 1979), females are poorly described and the morphology of males is still unknown. Because of this, we only compared a few characters of this species with our material; nonetheless, the following traits are sufficient to distinguish them: body size 8.44–9.1 mm in Sutton’s species vs. 18.35–21.41 mm in *P.* (*P*.) *baiomydis*; distance from the last spine to the tip of the tail (108 vs. 1660), and distribution (South America vs. North America).

Besides their distribution (Russia), *P.* (*P*.) *baicalensis* (Spassky, Ryzhikov and Sudarikov, 1952) and *P.* (*P*.) *sibiricensis* (Morozov, 1959) differ from the new species in having fewer prevulvar combs (31, 33–34, and 38–44, respectively). In addition, *P.* (*P*.) *baicalensis* has 18 bicuspid perioral denticles (instead of 25 unicuspid ones), and the shape of the oral opening of *P.* (*P*.) *sibiricensis* is rectangular, not hexagonal.

*Pterygodermatites* (*Paucipectines*) *baiomydis* is the 19th species of the subgenus described worldwide and the fourth collected in Mexico. Since cricetid rodents are one of the most speciose groups of mammals of the New World with approximately 600 species [25], its helminth fauna is incompletely documented. From the 141 cricetid species known to occur in Mexico [25], only 26 have been examined for helminths. The surveys of more species of this host group could result in the description of numerous new species of helminths, including representatives of *Pterygodermatites*.
